# Methods to increase response rates to a population-based maternity survey: a comparison of two pilot studies

**DOI:** 10.1186/s12874-019-0702-3

**Published:** 2019-03-20

**Authors:** Siân Harrison, Jane Henderson, Fiona Alderdice, Maria A. Quigley

**Affiliations:** Policy Research Unit in Maternal Health and Care, National Perinatal Epidemiology Unit, Nuffield Department of Population Health, University of Oxford, Old Road Campus, Oxford, OX3 7LF UK

**Keywords:** Survey, Questionnaire, Pregnancy, Maternity care, Postnatal care, Response rate

## Abstract

**Background:**

Surveys are established methods for collecting population data that are unavailable from other sources; however, response rates to surveys are declining. A number of methods have been identified to increase survey returns yet response rates remain low. This paper evaluates the impact of five selected methods on the response rate to pilot surveys, conducted prior to a large-scale National Maternity Survey in England.

**Methods:**

The pilot national maternity surveys were cross-sectional population-based questionnaire surveys of women who were three months postpartum selected at random from birth registrations. Women received a postal questionnaire, which they could complete on paper, online or verbally over the telephone. An initial pilot survey was conducted (pilot 1, *n* = 1000) to which the response rate was lower than expected. Therefore, a further pilot survey was conducted (pilot 2, *n* = 2000) using additional selected methods with the specific aim of increasing the response rate. The additional selected methods used for all women in pilot 2 were: pre-notification, a shorter questionnaire, more personable survey materials, an additional reminder, and inclusion of quick response (QR) codes to enable faster access to the online version of the survey. To assess the impact of the selected methods, response rates to pilot surveys 1 and 2 were compared.

**Results:**

The response rate increased significantly from 28.7% in pilot 1 to 33.1% in pilot 2 (+ 4.4%, 95%CI:0.88–7.83, *p* = 0.02). Analysis of weekly returns according to time from initial and reminder mail-outs suggests that this increase was largely due to the additional reminder. Most respondents completed the paper questionnaire rather than taking part online or over the telephone in both pilot surveys. However, the overall response to the online questionnaire almost doubled from 1.8% in pilot 1 to 3.5% in pilot 2, corresponding to an absolute difference of 1.7% (95%CI:0.45–2.81, *p* = 0.01), suggesting that QR codes might have facilitated online participation.

**Conclusions:**

Declining survey response rates may be ameliorated with the use of selected methods. Further studies should evaluate the effectiveness of each of these methods using randomised controlled trials and identify novel strategies for engaging populations in survey research.

**Electronic supplementary material:**

The online version of this article (10.1186/s12874-019-0702-3) contains supplementary material, which is available to authorized users.

## Background

Surveys are an important research method for collecting population data that are unavailable from other sources. However, response rates to surveys have been declining over the last 30 years [1]. The declining trend is exemplified in questionnaire surveys into maternal and infant health. For example, the response rate to the National Maternity Survey (NMS), which uses postal questionnaires to survey mothers during the postnatal period, has fallen from 67% in 1995 to 47% in 2014 [2–4]. This decline is consistent with other large UK maternity surveys, such as the Infant Feeding Surveys [5] and the Care Quality Commission Maternity Surveys [6].

A number of possible explanations have been proposed for the growing problem of non-response to survey research including greater time pressures, the increasing number of surveys in circulation, survey fatigue and privacy concerns [7]. There is a large literature reporting methods to improve survey response rates. A 2009 Cochrane review identified 481 trials that examined 110 different methods to increase response rates to postal and internet-based questionnaire surveys [8]. A number of strategies were found to substantially increase the odds of response to postal surveys including: offering incentives, pre-notifying individuals, shortening questionnaires, follow-up contact and employing more personable questionnaires. More recent research has consistently endorsed the use of incentives and multiple contacts [9–11] whereas evidence for pre-notification, shorter and more personable questionnaires is less clear [10–13]. Four methods (pre-notice letter, shorter questionnaire, postcard reminder, redesigned cover letter) were tested in a large randomised experiment conducted due to a persistent decline in response to the annual GP Patient Survey in 2015 [14]. With the exception of the pre-notice letter, all methods had a positive impact and, following implementation of these, the response rate to the GP Patient Survey increased, yet still remained below 40%.

Literature is emerging on methods to increase survey returns yet response rates remain low. If we are to continue to use postal and online questionnaire surveys to collect vital data on population health, the problem of declining response rates needs to be addressed. The aim of this paper is to evaluate the impact of five selected methods on the response rate to pilot surveys, conducted prior to a large-scale NMS in England.

## Methods

### Design and participants

The pilot surveys were cross-sectional population-based questionnaire surveys of postpartum women, administered via the postal system. The women were identified at random by the Office for National Statistics (ONS) using birth registration records. The sample included women aged 16 years and over who had given birth during specified one-week time-periods during July 2016 (pilot 1) or March 2017 (pilot 2). These time-periods were specified to ensure all women were three months postpartum at the time they were first contacted. The women were all living in England and the sample was stratified by region of residence (nine regions formerly known as the Government Office Regions [15]). Checks on infant deaths were made prior to the initial mail-out and any women whose babies had died were excluded from the sample and replacements were selected.

The initial pilot survey of 1000 women (pilot 1) was conducted to assess the survey procedures and materials ahead of the planned large-scale survey. The response rate to pilot 1 was lower than expected, based on response rates to previous NMS [2–4]. Therefore, the second pilot survey of 2000 women (pilot 2) was conducted using additional selected methods with the specific aim of increasing the response rate. Different women were sampled for the two pilot surveys. The sample size for pilot 2 was calculated to ensure sufficient power (80%) to detect a 5% increase in response (compared to that observed in pilot 1) as statistically significant.

Table 1 shows the study characteristics for pilot surveys 1 and 2. The principal methods employed in the pilot surveys were the same. However, additional selected methods were implemented in pilot 2. The selected methods were: 1) sending pre-notification cards prior to the initial questionnaire to inform the identified women they had been selected for the survey; 2) shortening the questionnaire from 20 pages (as in pilot 1) to 16 pages; 3) improving the design and content of the survey materials to make them more personable (specifically, changing the language used in the information that accompanied the questionnaire and changing the appearance of the questionnaire to make it more engaging following input from a design consultant and feedback from the advisory group and members of the target population); 4) sending one additional reminder (two in total); 5) including quick response (QR) codes on the questionnaires to enable easier access to the online questionnaire. The questionnaires developed for pilot survey 1 (Additional file 1) and pilot survey 2 (Additional file 2) are available separately.

**Table 1:** Study characteristics for pilot surveys 1 and 2

Pilot survey	1	2
Year of survey	2016	2017
Region	England (nine regions – former GOR*)	England (nine regions – former GOR*)
Number of women sampled	1000	2000
Period of birth	July 2016	March 2017
Baby age at recruitment	3 months	3 months
Pre-notification	No	Yes
Time of initial mail out	October 2016	June 2017
Modes of response available	Postal Telephone (interpretation service) Online	Postal Telephone (interpretation service) Online
Quick response (QR) codes	No	Yes
Number of reminders	1	2
Timing of reminders	+ 4 weeks: reminder questionnaire (week 5)	+ 4 weeks: reminder questionnaire (week 5) + 10 weeks: reminder questionnaire (week 11)
Length of questionnaire	20 pages	16 pages
Design of questionnaire	Based on previous National Maternity Surveys	More user-friendly language More engaging appearance

*Government Office Regions

Written questionnaire packs were mailed to the women by ONS and returned directly to the research team. Women were able to complete the questionnaire on paper, online or over the telephone by contacting the research team and answering the questions verbally (with an interpreter if required). The questionnaire asked women about their pregnancy, labour and birth, and postnatal care using predominantly structured questions with multiple-choice items and Likert scales for responses. The questionnaire also included some open questions allowing respondents to provide clarification on specific points and to express their views and describe their experiences in their own words if they wished. The questionnaire was split into ten sections with between 3 and 18 questions within each section. Reminder letters and additional questionnaires were mailed to non-respondents using a tailored reminder system [16].

### Statistical analysis

Baseline sociodemographic characteristics (age, marital status at the time of registering the birth of the baby, country of birth, level of area deprivation measured by the index of multiple deprivation (IMD), region of residence) were available from ONS for the women selected for the samples in pilot surveys 1 and 2. The baseline characteristics of each sample of women were described, and the differences were compared using Chi-square tests.

The cumulative response rates and the weekly response rates to pilot surveys 1 and 2 were compared graphically. The impact of the selected methods on the response rate was assessed by estimating the difference in the proportions and 95% confidence intervals (CI) of women who responded to pilot surveys 1 and 2. The differences in the proportions of women who had responded to pilot surveys 1 and 2 at specific time-points during the mail-out process (i.e. after initial and reminder mail-outs) and via the different modes (i.e. postal, online, telephone) were estimated, together with 95% CI. Finally, the differences in the response rates to pilot surveys 1 and 2 by women with different sociodemographic characteristics were estimated, together with 95% CI. The differences were compared using Chi-square tests.

## Results

Table 2 shows the baseline sociodemographic characteristics of the women selected for the samples in pilot surveys 1 and 2. Overall, there were no differences between the two samples of women in terms of age, marital status at the time of registering the birth of the baby, country of birth, IMD, or region of residence (*p* > 0.05).

**Table 2:** Baseline sociodemographic characteristics for pilot survey samples

	Pilot 1		Pilot 2		*p*-value
	N = 1000		*N* = 2000		
Maternal data	n	%	n	%	
Age (years)^a^					0.49
16–24	152	15.2	317	16.2	
25–29	268	26.8	559	28.6	
30–34	336	33.6	638	32.7	
35+	244	24.4	440	22.5	
Marital status at registration^a^					0.49
Married	524	52.4	1069	54.7	
Joint registration (same address)	337	33.7	607	31.1	
Joint registration (different address)	97	9.7	187	9.6	
Sole registration	42	4.2	91	4.7	
Country of birth^a^					0.54
UK	709	70.9	1364	69.8	
Outside UK	291	29.1	590	30.2	
Index of multiple deprivation (IMD)^a^					0.40
1st (most deprived)	250	25.0	494	25.3	
2nd	240	24.0	445	22.8	
3rd	209	20.9	368	18.8	
4th	151	15.1	337	17.2	
5th (least deprived)	150	15.0	310	15.9	
Region^a^					0.99
North East	49	4.9	83	4.2	
North West	138	13.8	265	13.6	
Yorkshire & the Humber	105	10.5	200	10.2	
East Midlands	85	8.5	160	8.2	
West Midlands	107	10.7	215	11.0	
East of England	113	11.3	223	11.4	
London	198	19.8	368	18.8	
South East	141	14.1	302	15.5	
South West	64	6.4	138	7.1	

^a^Sociodemographic data available for 1954 women from pilot survey 2 sample

**Fig. 1:**
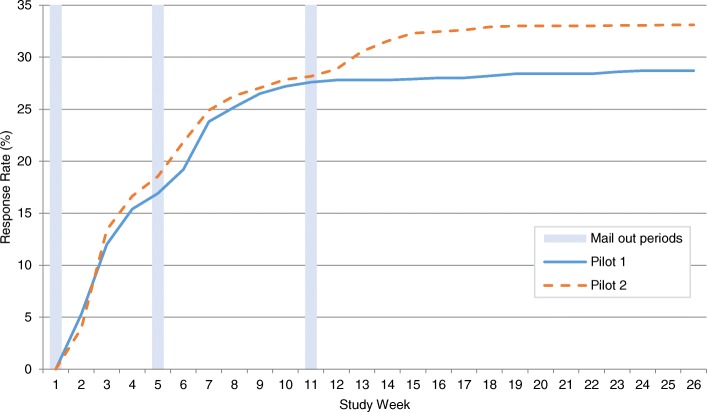
Cumulative weekly response rates to pilot surveys 1 and 2

Figure 1 shows the cumulative weekly response rates to pilot surveys 1 and 2 (additional file 3). The pattern of response was similar in both pilot surveys with pilot 1 response falling marginally behind pilot 2 response throughout most of the data collection period.

Table 3 shows the overall response rates to pilot surveys 1 and 2 and the difference between the response rates with 95% CI. The response rate to pilot 1 at the end of the survey was 28.7% and the response rate to pilot 2 at the end of the survey was 33.1%, which represents a 4.4% increase in response (95%CI:0.88 to 7.83, *p* = 0.02).

**Table 3:** Response rates to pilot surveys 1 and 2

	Pilot 1 (N = 1000)		Pilot 2 (N = 2000)		% difference	95% CI for % difference	*p*-value
	n^	%	n^	%			
After initial mail out (Before reminder 1 mail out)	169	16.9	371	18.6	+1.7	-1.25, 4.52	0.25
After reminder 1 mail out (Before reminder 2 mail out)	276	27.6	564	28.2	+0.6	-2.84, 3.95	0.73
End of survey (After reminder 2 mail out in pilot 2 only)	287	28.7	662	33.1	+4.4	0.88, 7.83	0.02*
Postal (end of survey)	268	26.8	593	29.7	+2.9	−0.54, 6.25	0.10
Online (end of survey)	18	1.8	69	3.5	+1.7	0.45, 2.81	0.01*
Telephone (end of survey)	1	0.1	0	0	−0.1	−0.11, 0.56	0.16

*Chi-square significant at *p* < 0.05

^ Number of responses

**Fig. 2:**
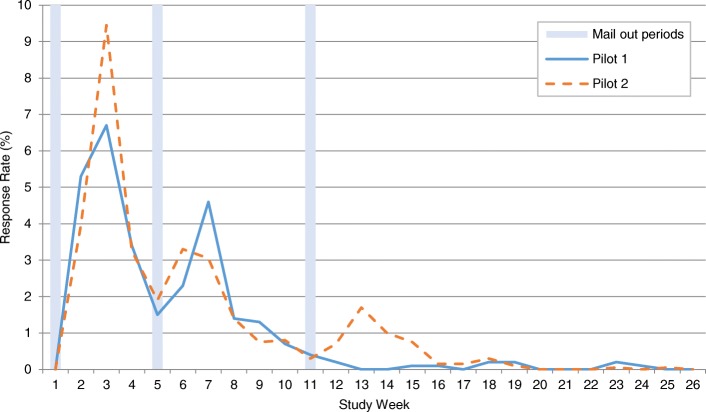
Weekly response rates to pilot surveys 1 and 2

Figure 2 shows the percentage of responses received during each week of pilot surveys 1 and 2 (additional file 4). There were increases in response following each of the mail-out periods. The response rate was highest to the initial mail-out in both pilots with a diminished return after each subsequent mail-out.

Table 3 shows a breakdown of the response rates to each pilot survey by different time-points during the mail-out process. After the initial mail-out and prior to the first reminder, the response rate to pilot 2 was 1.7% higher than the response rate to pilot 1 (95%CI: -1.25 to 4.52, *p* = 0.25). After the first reminder and prior to the second reminder (in pilot 2 only), the response rate to pilot 2 was 0.6% higher than to pilot 1 (95%CI: -2.84 to 3.95, *p* = 0.73). Therefore, at these equivalent time-points in the mail-out process in pilot surveys 1 and 2, there were small increases in response to pilot 2 which were not statistically significant.

Table 3 also shows a breakdown of the response rates to each pilot survey by mode of response. In pilot 1, the majority of women opted to complete and return the postal questionnaire (26.8%) rather than to take part online (1.8%) or over the telephone (0.1%). The preference for taking part via post was also evident in pilot 2 with 29.7% opting to complete and return the postal questionnaire compared to 3.5% opting to take part online; no women utilised the telephone option in pilot 2. Although the numbers are small, the proportion of women choosing to take part online almost doubled from 1.8% in pilot 1 to 3.5% in pilot 2, which corresponds to an absolute increase of 1.7% (95%CI: 0.45 to 2.81, *p* = 0.01).

Table 4 shows a breakdown of the response rates to each pilot survey according to maternal sociodemographic characteristics. There was some evidence of a larger increase in the response rate to pilot 2 compared to pilot 1 by women with certain sociodemographic characteristics, for example, a higher proportion of women aged 30–34 years, married women, and women born in the UK and outside of the UK responded in pilot survey 2. However, the numbers of women included in these subgroup analyses were small and the confidence intervals for the differences overlap.

**Table 4:** Response rates to pilot surveys 1 and 2 by maternal sociodemographic characteristics

	Pilot 1 (*N* = 287)		Pilot 2 (*N* = 662)		% difference	95% CI for % difference
	n^	%^+^	n^	%^+^		
Age (years)						
16–24	28	18.4	57	18.0	−0.4	− 6.7, 8.3
25–29	69	25.7	159	28.4	+2.7	−3.9, 8.9
30–34	95	28.3	252	39.5	+11.2	4.9, 17.2
> = 35	95	38.9	194	44.1	+5.2	−2.5, 12.7
Marital status						
Married	175	33.4	452	42.3	+8.9	3.8, 13.8
Joint registration (same address)	90	26.7	166	27.3	+0.6	−5.4, 6.4
Joint registration (different address)	15	15.5	32	17.1	+1.6	−8.1, 10.0
Sole registration	7	16.7	12	13.2	−3.5	−8.4, 18.5
Country of birth						
UK	228	32.2	500	36.7	+4.5	0.2, 8.7
Not UK	59	20.3	162	27.5	+7.2	1.1, 12.8
Index of multiple deprivation (IMD)						
1st (most deprived)	40	16.0	104	21.1	+5.1	−1.0, 10.6
2nd	59	24.6	130	29.2	+4.6	−2.5, 11.3
3rd	66	31.6	125	34.0	+2.4	−5.7, 10.1
4th	61	40.4	147	43.6	+3.2	−6.3, 12.4
5th (least deprived)	61	40.7	156	50.3	+9.6	−0.1, 18.9
Region						
North East	17	34.7	27	32.5	−2.2	−13.7, 18.9
North West	35	25.4	85	32.1	+6.7	−2.8, 15.5
Yorkshire & the Humber	23	21.9	65	32.5	+10.6	−0.1, 20.2
East Midlands	22	25.9	48	30.0	+4.1	−8.0, 15.2
West Midlands	29	27.1	74	34.4	+7.3	−3.6, 17.3
East of England	39	34.5	83	37.2	+2.7	−8.3, 13.1
London	51	25.8	104	28.3	+2.5	−5.3, 9.9
South East	51	36.2	122	40.4	+4.2	−5.6, 13.5
South West	20	31.3	54	39.1	+7.8	−6.6, 20.8

^ Number of responses

^+^Response rate within subcategory

The analysis focused on unit non-response but item non-response was also assessed. The proportion of missing data was below 5% for all key non-optional items on the questionnaire.

## Discussion

In this study, a number of methods were evaluated for increasing response rates in an English population-based maternity questionnaire survey. Taken together, pre-notification, a shorter questionnaire, more personable study materials, an additional reminder and the inclusion of QR codes led to an increase in the response rate. Although the overall increase was modest, it was statistically significant and methodologically important against a persistent downward trend in response rates to surveys. The findings show that declining response rates may be ameliorated with the use of these selected survey methods. There was some evidence to suggest that the selected methods may have had a greater impact on women with certain sociodemographic characteristics, although the numbers of women included in the subgroup analyses were small. Further research is required to explore how different research methods might affect response rates in different sociodemographic groups.

The methods evaluated in this study were mostly found to have a limited effect on the response rate but, due to the timing of responses, the findings suggest that reminders might be important. The literature on survey methods confirms the use of multiple contacts as one of the most influential factors in improving questionnaire returns [8, 17]. Despite this finding, objections to contacting potential research participants on numerous occasions are sometimes raised on ethical grounds. Clearly it is essential to protect potential participants from feeling coerced into taking part in studies. However, we also have a responsibility to those individuals who do choose to participate. Quality research requires the recruitment of sufficient participants to address the research questions and to draw valid inferences from the data collected. A 2008 synopsis of best practices for survey research recommended ‘when conducting survey research, if follow-ups are not planned and budgeted for, the study should not be initiated’ (p.2) [17].

The other method that was shown to be potentially effective in this study was the use of QR codes to enable easier access to the online questionnaire. Although the number of women opting to take part via the online survey was small and the use of this method did not significantly impact the overall response rate, the online response rate almost doubled when they were included. Therefore, the use of QR codes may have facilitated participation via this mode. According to the marketing literature, QR codes are simple and effective tools which increase user engagement [18]. However, there is very little information in the academic literature to support or refute the effectiveness of this technology.

Other methods that have previously been shown to increase response rates to questionnaire surveys were not found to be effective in this study. Our finding that pre-notification did not have a significant effect on response is consistent with the GP Patient Survey experiment [14] yet contrary to Cochrane review evidence [8]. The evidence for the impact of questionnaire length on response rate is unclear, with some studies suggesting that response rates improve with shorter questionnaires [8, 19] and others suggesting that response rate is unrelated to questionnaire length [10, 11]. This study did not provide support for using shorter questionnaires, although it is possible that, despite shortening the questionnaire, it was still deemed to be too long [20]. Finally, using more personable study materials did not increase the response rate in this study, contrary to existing findings, which suggest that improving study materials is an effective method [8]. However, the literature is not clear on what exactly constitutes respondent friendly design; hence the extent to which the material was improved in this study is uncertain.

According to the literature, the most effective method to increase response rates to surveys is to offer incentives to participants [8–11]. However, as with multiple contacts, the use of incentives raises ethical considerations [21]. Traditionally, offering remuneration to individuals for their involvement in research has been discouraged due to concerns around coercion [22], yet the use of incentives is now becoming increasingly recognised as an acceptable, and often necessary, strategy to aid recruitment. Furthermore, remuneration may be an indication of respect for the time and contribution that research participants make [23]. Nonetheless, offering incentives is not without issues; it may generate selection bias, undermine autonomy around consent, preclude participant anonymity, and substantially increase the cost of research studies [21]. Indeed, we were unable to offer incentives in this study due to limited resources and budgeting for incentives in similar large-scale surveys might not always be feasible.

The main strength of this study is that a statistically significant increase in response rate was achieved with the implementation of selected methods aimed at increasing response. This increase was achieved over a period of eight months against a downward trend in response rates to postal questionnaire surveys of maternal and infant health over more than 30 years [2–6]. The methods shown to be potentially effective in this study can now be developed and incorporated into future population-based maternity surveys. Another strength is the consistency in the design and materials employed in the two pilot surveys enabling direct comparison of the results.

The main limitation is that women were not randomised to one of the two pilot surveys. However, the women selected for both pilots were random samples from the population of all births and comparison of the women indicated they did not differ on key sociodemographic characteristics. Another limitation is that we were unable to isolate the impact of the different methods, possibly with the exception of the additional reminder. Due to the fact that pilot surveys 1 and 2 were not carried out at the same time of year, it is also possible that factors we were unable to control for might have impacted upon the response rates. For example, pilot 1 was launched during the Autumn (Fall) and ran through the Winter whereas pilot 2 was launched during the Spring and ran through the Summer. Consequently, the climatic conditions and the holiday periods during the two pilots would have been different and seasonal effects might have affected the response rate.

## Conclusions

Declining response rates may be ameliorated with the use of selected survey methods. Additional evidence from randomised controlled trials is required to offer clearer guidance on which methods are most effective for maximising postal and online questionnaire survey returns. Further research is also required to identify novel strategies for engaging populations in survey research.

## Additional files

Additional file 1: A national survey of mothers and babies: maternity, health and care. Pilot survey 1 questionnaire. (PDF 1623 kb)

Additional file 2: You and Your Baby: A national survey of health and care. Pilot survey 2 questionnaire. (PDF 1852 kb)

Additional file 3: **Figure S1.** Line graph to show the cumulative weekly response rates to pilot surveys 1 and 2 in relation to the mail out periods. (DOCX 194 kb)

Additional file 4: **Figure S2.** Line graph to show the weekly response rates to pilot surveys 1 and 2 in relation to the mail out periods. (DOCX 189 kb)

## Abbreviations

CI: Confidence interval
NMS: National Maternity Survey
ONS: Office for National Statistics
QR: Quick response
