# Supplementary material for: Methods to increase response rates to a population-based maternity survey: a comparison of two pilot studies
Source: BMC Med Res Methodol. 2019 Mar 20;19:65. doi: 10.1186/s12874-019-0702-3 (PMC6425628; doi:10.1186/s12874-019-0702-3)
Supplement: Supplementary file 1 — A national survey of mothers and babies: maternity, health and care. Pilot survey 1 questionnaire. (PDF 1623 kb) [file 12874_2019_702_MOESM1_ESM.pdf]

Questionnaire 1.1, 16-Sept-2016

# A national survey of mothers and babies: maternity, health and care

# 2016

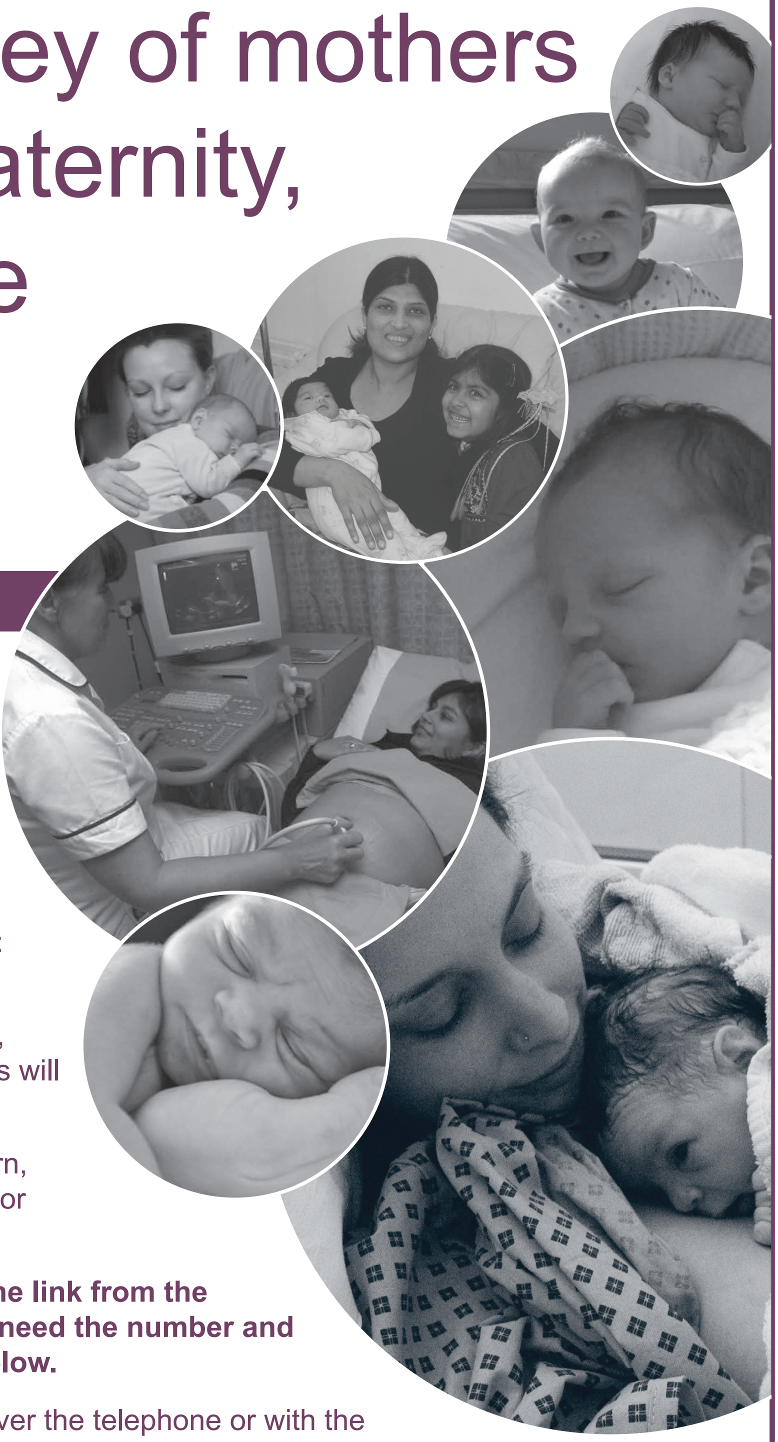

This is an independent survey about your recent experience of pregnancy, childbirth and the health of you and your baby. It is important to hear the views of as many women as possible.

We asked the Office for National Statistics (ONS) to send out this survey on our behalf. They have **not** told us your name and we **do not** have access to any of your details.

If you would prefer not to fill in this questionnaire, please just return it in the pre-paid envelope. This will ensure that we don't contact you again.

If the survey raises questions or issues of concern, you may wish to contact your family doctor (GP) or Health Visitor.

**You can complete this survey on-line using the link from the NPEU website (www.npeu.ox.ac.uk). You will need the number and password in your letter or in the small box below.**

If you would like to complete the questionnaire over the telephone or with the help of a Language Line interpreter, please telephone this Freephone number 0808 252 4566.

National Perinatal Epidemiology Unit
University of Oxford
Old Road Campus
Headington
Oxford OX3 7LF

PRUMHC
POLICY RESEARCH UNIT - MATERNAL HEALTH & CARE

npeu
National Perinatal Epidemiology Unit

# How to complete this questionnaire

- If you have been pregnant before, please only think about the maternity care you received in your **most recent pregnancy and birth** when answering these questions.

- For most questions, make a cross clearly inside one box ☒ or write clearly in the space provided. For some questions where indicated you may mark more than one box.

- Not all sections will apply to you. We estimate that the questionnaire will take around 30 minutes to complete.

## Section A: Dates and Your Baby

**A1 Did you give birth to a single baby, twins or more in your most recent pregnancy?**

☐1 A single baby ☐2 Twins ☐3 Triplets, quads or more

**A2 If you had a single baby this time, is your baby … ?** *Please tick one box*

☐1 A boy **OR** ☐2 A girl

**If you had a multiple birth, did you have…?**

☐3 All boys **OR** ☐4 All girls **OR** ☐5 boy and girl twins, triplets or more

**Note:** If you had more than one baby in your most recent pregnancy, please fill in the rest of the questionnaire about the baby who was born first as a result of this pregnancy

**A3 When was your baby born?**

D D / M M / Y Y (date / month / year)

**A4 Roughly how many weeks pregnant were you when your baby was born?**

☐☐ weeks

**A5 How much did your baby weigh at birth?** *Please remember if you had more than one baby, to fill in this questionnaire about the baby who was born first*

**Either** What your baby weighed in grams ☐☐☐☐

**Or** What your baby weighed in pounds ☐☐ and ounces ☐☐

## Section B: Antenatal Care

**B1 Did you plan to get pregnant with this baby?** ☐1 Yes ☐2 No

**B2 How would you describe your reaction when you realised you were pregnant with this baby?** *Please tick one box only*

☐1 Overjoyed
☐2 Pleased
☐3 Mixed feelings
☐4 A bit unhappy
☐5 Very unhappy
☐6 No particular feelings

**B3 Roughly how many weeks pregnant were you when you had your pregnancy 'booking'?** *(the appointment where you were given your hand held pregnancy records / notes)*

☐☐ weeks

**B4 Around the time of your pregnancy booking were you asked about your emotional and mental health?**

☐1 Yes ☐2 No

**B5 Around the time of your pregnancy booking were you asked about your past mental health or family history of mental health (before this pregnancy)?**

☐1 Yes ☐2 No

## Antenatal check-ups

A 'check-up' is any contact with a midwife or a doctor to check the progress of your pregnancy. This usually includes having your blood pressure and urine checked. *Please ignore other appointments that did not include these things, such as a visit for a scan or a blood test only.*

**B6 During your pregnancy did you have any antenatal check-ups?**

☐ Yes ☐ No ☐ Not sure / Don't know

**B7 Roughly how many check-ups did you have at each of these places?**
*Please write in the number for each*

Local clinic ☐☐
Children's centre ☐☐
GP surgery ☐☐
Hospital clinic ☐☐
At home ☐☐
Other place ☐☐ *Please give details* ____________________

**B8 Altogether, how many different midwives looked after you during your pregnancy?**
*Please tick one box only*

☐ One ☐ Two ☐ Three ☐ Four ☐ Five or more

**B9 During your pregnancy did you have a health professional you could talk to about personal or sensitive issues?**

☐ Yes, always ☐ Yes, to some extent ☐ No

**B10 During your pregnancy, before you went into labour did you stay in hospital overnight?**
*(**not** including admission for induction or before planned caesarean section)*

☐ Yes ☐ No

**B11 Before you went into labour or had your baby did you have any particular worries about the labour and birth?** *Please tick one box for each line*

| | Very worried | Quite worried | Not very worried | Not at all worried |
|---|---|---|---|---|
| Not knowing when I would go into labour | ☐ | ☐ | ☐ | ☐ |
| Getting to the hospital in time | ☐ | ☐ | ☐ | ☐ |
| Having to be induced | ☐ | ☐ | ☐ | ☐ |
| Having a long labour | ☐ | ☐ | ☐ | ☐ |
| Pain and discomfort of labour | ☐ | ☐ | ☐ | ☐ |
| Getting effective pain relief | ☐ | ☐ | ☐ | ☐ |
| Not knowing how long labour would take | ☐ | ☐ | ☐ | ☐ |
| Having a forceps or ventouse delivery | ☐ | ☐ | ☐ | ☐ |
| Embarrassment | ☐ | ☐ | ☐ | ☐ |
| Needing a caesarean | ☐ | ☐ | ☐ | ☐ |
| Other *Please give details* ____________ | ☐ | ☐ | ☐ | ☐ |

## During your pregnancy

**B12 Did you have any long term health problems which made your pregnancy difficult or complicated?** *(e.g. epilepsy or diabetes)*

☐ Yes ☐ No *Please give details*

**B13 Did you have specific pregnancy related problems which affected you or your baby?** *(e.g. high blood pressure, threatened preterm labour, low-lying placenta)*

☐ Yes ☐ No *Please give details*

# Section C: Your Labour and the Birth of your Baby

**C1 Where was your baby born?** *Please tick one box only*

- ☐ In hospital, in a midwife-led unit
- ☐ In hospital, in a consultant-led unit
- ☐ In a midwife-led unit or birth centre separate from hospital
- ☐ At home
- ☐ Other

**C2 Did you have a labour?** ☐ Yes ☐ No **If No, *please go to question C7***

**C3 How did your labour start?** *Please tick all that apply*

- ☐ It started naturally
- ☐ I had one or more membrane sweeps
- ☐ I was given a vaginal gel or pessary to induce my labour
- ☐ My waters were broken by a doctor or a midwife (amniotomy)
- ☐ I was given a drip (in my hand or arm) to induce my labour

**C4 Roughly how long did your labour last?**

☐☐ hours **AND / OR** ☐☐ minutes

**C5 During your labour, did you use any of the following to relieve the pain?**
*Please tick all that apply*

- ☐ Gas and air *(breathing through a mask)*
- ☐ Injection of pethidine or a similar painkiller
- ☐ Epidural or similar *(injection in your back)*
- ☐ Other *Please give details* ______________________

**C6 During your labour, how was your baby monitored?** *Please tick all that apply*

- ☐ Staff listened with a 'pinard' (ear trumpet) now and then
- ☐ Sonicaid (a hand held monitor) was used now and then
- ☐ A monitor was used now and then, with a belt around my tummy
- ☐ A monitor was used constantly with a belt around my tummy
- ☐ A monitor was used constantly with a clip attached to my baby's head
- ☐ I had no monitoring / Not sure / Can't remember

## The birth of your baby

**C7 Thinking about the birth of your baby, what kind of delivery did you have?** *If you had more than one baby, please answer about the baby who was born first. Please tick one box only*

- ☐ 1 Normal (vaginal) birth
- ☐ 2 Delivery using forceps
- ☐ 3 Delivery using vacuum cap on the baby's head (ventouse)
- ☐ 4 A caesarean (through a cut in the abdomen)

## Caesarean Birth

**Note:** If you did **not** have a caesarean for this birth, ***please go to question C9***

**C8 If your baby was born by caesarean this time was this…** *Please tick one box only*

- ☐ 1 Planned and carried out before you went into labour?
- ☐ 2 Planned, but carried out after you had gone into labour?
- ☐ 3 The result of an unforeseen problem during your labour?

## Vaginal Birth

**C9 If you had a vaginal birth, while your baby was being born did you have an episiotomy (cut) to assist with the delivery of the baby?**

☐ 1 Yes ☐ 2 No ☐ 3 Don't know / Can't remember

**C10 If you had a vaginal birth, while your baby was being born did you have a tear (not a deliberate cut)?** *Please tick one box only*

- ☐ 1 No
- ☐ 2 Yes, a tear that did not need stitches
- ☐ 3 Yes, a tear that needed stitches
- ☐ 4 Yes, a serious tear which involved my back passage (third or fourth degree tear)
- ☐ 5 Don't know / Can't remember

## The staff caring for you during labour and birth

Please answer the following section if you had a vaginal birth **or** a caesarean.

**C11 Altogether, how many different midwives looked after you during your labour and / or the birth of your baby?** *Please tick one box only*

☐ 1 One ☐ 2 Two ☐ 3 Three ☐ 4 Four ☐ 5 Five or more

**C12 Had you met any of these midwives before you went into labour or gave birth?** *Please tick one box only*

☐ 1 All of them ☐ 2 Some of them ☐ 3 None of them

**C13 How soon after birth did you…**

Hold your baby? ☐☐ minutes ☐☐ hours ☐☐ days

Have skin-to-skin contact with your baby? ☐☐ minutes ☐☐ hours ☐☐ days

**C14 We would like to know how you feel you were looked after during your labour and birth. Please circle as many words as you like which describe the staff you saw during labour.**

| | | | |
|---|---|---|---|
| rushed | humorous | insensitive | kind |
| considerate | unhelpful | supportive | offhand |
| rude | warm | inconsiderate | polite |
| sensitive | bossy | informative | condescending |

**C15 Overall, how do you feel your labour and birth went?** *Please tick one box only*

☐ Worse than you expected ☐ More or less as you expected ☐ Better than you expected

## Section D: You and Your Baby After the Birth

**D1 Did you stay in a maternity unit (or hospital) after your baby was born?**

☐ Yes ☐ No

**D2 If Yes, how long did you stay in the maternity unit (or hospital) after your baby was born?**

☐☐ hours OR ☐☐ days ☐ Does not apply

After birth some babies need specialist care, e.g. help with breathing, and are admitted to a neonatal unit, sometimes called a Special Care Baby Unit (SCBU) or Neonatal Intensive Care Unit (NICU).

**D3 Was your baby cared for in a neonatal unit at all?** ☐ Yes ☐ No

If Yes, for how long? ☐☐ days **OR** ☐☐ weeks

**D4 After the birth of your baby, how many times in total did you….**

See a midwife at home? ☐☐ times

See a midwife at drop in clinic? ☐☐ times

See a maternity support worker at home? ☐☐ times

Contact a midwife or maternity support worker by phone? ☐☐ times

**D5 How old was your baby when you had the last visit or contact with the midwife or maternity support worker?**

Age in ☐☐ days **OR** ☐☐ weeks

**D6 How many different midwives in total visited you at home after your baby was born?** *Please tick one box only*

☐ None ☐ One ☐ Two ☐ Three or more

**D7 Had you met any of these midwives before you had your baby?** *Please tick one box only*

☐ All of them ☐ Some of them ☐ None of them

**D8 Since your baby was born have you been asked about your emotional and mental health by a health professional?**

☐ Yes ☐ No

**D9 Did you have a postnatal check-up of your own health with your GP (family doctor), usually between 4-8 weeks after the birth?**

☐ Yes ☐ No

## Feeding Your Baby

**Note:** When we ask about 'breastfeeding' we also mean 'giving your baby expressed breast milk'

**D10 Did you ever try to breastfeed your baby, even if it was only once?**

- [ ] Yes
- [ ] No

**D11 In the first few days after the birth how was your baby fed?** *Please tick one box only*

- [ ] Formula (bottle) milk only
- [ ] Breast milk (or expressed breast milk) only
- [ ] Both breast and formula (bottle) milk
- [ ] Not sure / can't remember

**D12 Who helped or advised you with feeding your baby?** *Please tick all that apply*

- [ ] Midwife
- [ ] Health visitor
- [ ] Other health professional
- [ ] Partner / friend / relative
- [ ] Breastfeeding support group
- [ ] Peer supporter (other mum who has breastfed, trained to give support to other mums)
- [ ] Voluntary organisation
- [ ] Online support / social media
- [ ] DVDs / books / magazines
- [ ] I was not given any help or advice
- [ ] I did not need any help or advice

**D13 Thinking about the milk that your baby has received over the last 7 days, has he or she had...?** *Please tick one box only*

- [ ] Only infant formula / other milk
- [ ] Only breast milk
- [ ] Both breast milk and infant formula / other milk
- [ ] Other *Please give details* ____________________

**D14 Would you have liked more help from a health professional with feeding your baby?**

- [ ] Yes
- [ ] No

**D15 If you breastfed, did you do so for as long as you wanted to?**

- [ ] Yes
- [ ] No
- [ ] Does not apply

**D16 How old was your baby when he or she was last given breast milk?**

[ ][ ] days **OR** [ ][ ] weeks [ ] My baby was never given breast milk

**D17 Has your baby ever had any solid foods such as cereal, rusks, baby rice, fruit, vegetables or any other kind of solid food?**

- [ ] Yes
- [ ] No

**D18 How old was your baby when he or she first had any solid food apart from milk?**

[ ][ ] days **OR** [ ][ ] weeks

## Section E: Your Experience of Maternity Care

**E1 Each woman's experience of maternity care is different. How much do you agree with the following statements?** *Please tick one box for each row*

| **Thinking about my care during pregnancy….** | **Strongly agree** | **Agree to some extent** | **Neither agree or disagree** | **Disagree to some extent** | **Strongly disagree** |
|---|---|---|---|---|---|
| I felt I had the right number of antenatal checks with a midwife / doctor | ☐ 1 | ☐ 2 | ☐ 3 | ☐ 4 | ☐ 5 |
| I did not have enough choice about my care during pregnancy *(e.g. who you saw, where and when)* | ☐ 1 | ☐ 2 | ☐ 3 | ☐ 4 | ☐ 5 |
| My care provider(s) gave me all the information I needed | ☐ 1 | ☐ 2 | ☐ 3 | ☐ 4 | ☐ 5 |
| I always saw the same midwife / doctor for my antenatal checks | ☐ 1 | ☐ 2 | ☐ 3 | ☐ 4 | ☐ 5 |
| I was not able to contact my midwife or other health professional when I needed to | ☐ 1 | ☐ 2 | ☐ 3 | ☐ 4 | ☐ 5 |
| I was not always treated with respect and kindness by health professionals | ☐ 1 | ☐ 2 | ☐ 3 | ☐ 4 | ☐ 5 |
| Health professionals did not always talk to me in a way I could understand | ☐ 1 | ☐ 2 | ☐ 3 | ☐ 4 | ☐ 5 |
| Antenatal appointments were too short to discuss any concerns about my pregnancy | ☐ 1 | ☐ 2 | ☐ 3 | ☐ 4 | ☐ 5 |
| I was able to speak to a health professional about any worries or sensitive issues | ☐ 1 | ☐ 2 | ☐ 3 | ☐ 4 | ☐ 5 |
| I was not involved enough in decisions about my antenatal care | ☐ 1 | ☐ 2 | ☐ 3 | ☐ 4 | ☐ 5 |
| I felt listened to when I talked to my care provider about my pregnancy and birth | ☐ 1 | ☐ 2 | ☐ 3 | ☐ 4 | ☐ 5 |
| I was happy with the number of health professionals who cared for me during my pregnancy | ☐ 1 | ☐ 2 | ☐ 3 | ☐ 4 | ☐ 5 |
| I was not given enough explanations about antenatal scans and tests | ☐ 1 | ☐ 2 | ☐ 3 | ☐ 4 | ☐ 5 |
| I was not given enough information to make decisions about my antenatal care | ☐ 1 | ☐ 2 | ☐ 3 | ☐ 4 | ☐ 5 |
| Seeing different midwives / doctors for antenatal care did not matter to me | ☐ 1 | ☐ 2 | ☐ 3 | ☐ 4 | ☐ 5 |
| I would have liked more antenatal checks and scans | ☐ 1 | ☐ 2 | ☐ 3 | ☐ 4 | ☐ 5 |
| During pregnancy I was given enough information about where I could give birth to my baby *(e.g. home, hospital, midwife unit)* | ☐ 1 | ☐ 2 | ☐ 3 | ☐ 4 | ☐ 5 |
| Health professionals always treated me as an individual | ☐ 1 | ☐ 2 | ☐ 3 | ☐ 4 | ☐ 5 |
| During pregnancy, I did not feel well cared for by health professionals | ☐ 1 | ☐ 2 | ☐ 3 | ☐ 4 | ☐ 5 |
| Overall, I was very pleased with the care I received in pregnancy | ☐ 1 | ☐ 2 | ☐ 3 | ☐ 4 | ☐ 5 |

## During labour and birth

**E2 How much do you agree with the following statements?** *Please tick one box for each row*

| **Thinking about my care during <u>labour and birth</u> ...** | **Strongly agree** | **Agree to some extent** | **Neither agree or disagree** | **Disagree to some extent** | **Strongly disagree** |
|---|---|---|---|---|---|
| Before my labour and birth I was well informed by my midwife / doctor about what would happen | ☐ 1 | ☐ 2 | ☐ 3 | ☐ 4 | ☐ 5 |
| Staff communicated well with me during labour and birth | ☐ 1 | ☐ 2 | ☐ 3 | ☐ 4 | ☐ 5 |
| My choices for labour and birth were not always respected | ☐ 1 | ☐ 2 | ☐ 3 | ☐ 4 | ☐ 5 |
| I needed more staff support during labour and birth | ☐ 1 | ☐ 2 | ☐ 3 | ☐ 4 | ☐ 5 |
| Everything was explained to me well during labour and birth | ☐ 1 | ☐ 2 | ☐ 3 | ☐ 4 | ☐ 5 |
| I was treated as an individual by staff | ☐ 1 | ☐ 2 | ☐ 3 | ☐ 4 | ☐ 5 |
| I was not involved enough in decisions about procedures that were carried out *(e.g. breaking waters, epidural, caesarean section)* | ☐ 1 | ☐ 2 | ☐ 3 | ☐ 4 | ☐ 5 |
| Health professionals left me alone more than I would have liked | ☐ 1 | ☐ 2 | ☐ 3 | ☐ 4 | ☐ 5 |
| I felt that my pain relief needs were not managed well | ☐ 1 | ☐ 2 | ☐ 3 | ☐ 4 | ☐ 5 |
| I had already met the staff who looked after me during labour and birth | ☐ 1 | ☐ 2 | ☐ 3 | ☐ 4 | ☐ 5 |
| I felt safe in the labour and birth environment | ☐ 1 | ☐ 2 | ☐ 3 | ☐ 4 | ☐ 5 |
| Staff did not listen to my requests in managing my labour and birth | ☐ 1 | ☐ 2 | ☐ 3 | ☐ 4 | ☐ 5 |
| The staff could have done more to help me to feel in control of my labour and birth | ☐ 1 | ☐ 2 | ☐ 3 | ☐ 4 | ☐ 5 |
| I had confidence and trust in the staff caring for me | ☐ 1 | ☐ 2 | ☐ 3 | ☐ 4 | ☐ 5 |
| Health professionals helped make labour and birth a really positive experience | ☐ 1 | ☐ 2 | ☐ 3 | ☐ 4 | ☐ 5 |
| The personal care I received could have been better during labour and birth | ☐ 1 | ☐ 2 | ☐ 3 | ☐ 4 | ☐ 5 |
| I did not mind being looked after by midwives or doctors I had not met before | ☐ 1 | ☐ 2 | ☐ 3 | ☐ 4 | ☐ 5 |
| The choices I wanted were not available to me | ☐ 1 | ☐ 2 | ☐ 3 | ☐ 4 | ☐ 5 |
| My labour and birth experience was not as I expected | ☐ 1 | ☐ 2 | ☐ 3 | ☐ 4 | ☐ 5 |
| I had the best possible care during labour and birth | ☐ 1 | ☐ 2 | ☐ 3 | ☐ 4 | ☐ 5 |

## After the birth

**E3 How much do you agree with the following statements?** *Please tick one box for each row*

| **Thinking about my postnatal care …** | **Strongly agree** | **Agree to some extent** | **Neither agree or disagree** | **Disagree to some extent** | **Strongly disagree** |
|---|---|---|---|---|---|
| I received enough care and attention from staff on the postnatal ward | ☐ 1 | ☐ 2 | ☐ 3 | ☐ 4 | ☐ 5 |
| I stayed in hospital as long as I wanted after the birth | ☐ 1 | ☐ 2 | ☐ 3 | ☐ 4 | ☐ 5 |
| Staff on the postnatal ward did not respond when I needed them | ☐ 1 | ☐ 2 | ☐ 3 | ☐ 4 | ☐ 5 |
| I was not able to make choices about the postnatal care of me and my baby | ☐ 1 | ☐ 2 | ☐ 3 | ☐ 4 | ☐ 5 |
| I was treated as an individual by midwives / doctors after the birth | ☐ 1 | ☐ 2 | ☐ 3 | ☐ 4 | ☐ 5 |
| After I had given birth, health professionals treated me as though I was no longer important | ☐ 1 | ☐ 2 | ☐ 3 | ☐ 4 | ☐ 5 |
| I did not know the midwives I saw after bringing my baby home | ☐ 1 | ☐ 2 | ☐ 3 | ☐ 4 | ☐ 5 |
| I had enough information from health professionals about how to care for my baby | ☐ 1 | ☐ 2 | ☐ 3 | ☐ 4 | ☐ 5 |
| I felt comfortable speaking with healthcare professionals about how I was feeling | ☐ 1 | ☐ 2 | ☐ 3 | ☐ 4 | ☐ 5 |
| The health professionals I saw after the birth did not really listen to me | ☐ 1 | ☐ 2 | ☐ 3 | ☐ 4 | ☐ 5 |
| I would have liked to have seen midwives more after the birth | ☐ 1 | ☐ 2 | ☐ 3 | ☐ 4 | ☐ 5 |
| I was able to build a good relationship with the healthcare professional(s) I saw after coming home | ☐ 1 | ☐ 2 | ☐ 3 | ☐ 4 | ☐ 5 |
| I was not given the advice and information I needed by health professionals after my baby was born | ☐ 1 | ☐ 2 | ☐ 3 | ☐ 4 | ☐ 5 |
| There was not enough time to talk over my concerns with health professionals | ☐ 1 | ☐ 2 | ☐ 3 | ☐ 4 | ☐ 5 |
| The advice I received from healthcare professionals about caring for my baby was consistent | ☐ 1 | ☐ 2 | ☐ 3 | ☐ 4 | ☐ 5 |
| I had all the checks I needed after the birth | ☐ 1 | ☐ 2 | ☐ 3 | ☐ 4 | ☐ 5 |
| After the birth of my baby, I knew who to contact if I had questions or concerns | ☐ 1 | ☐ 2 | ☐ 3 | ☐ 4 | ☐ 5 |
| As a mother of a new baby I did not feel cared for and supported enough by health professionals | ☐ 1 | ☐ 2 | ☐ 3 | ☐ 4 | ☐ 5 |
| The postnatal care I received did not meet the needs of me and my baby | ☐ 1 | ☐ 2 | ☐ 3 | ☐ 4 | ☐ 5 |
| Overall I was very pleased with the quality of my postnatal care | ☐ 1 | ☐ 2 | ☐ 3 | ☐ 4 | ☐ 5 |

## Section F: Your Baby

**F1 When did you first feel your baby really belonged to you?** *Please tick one box only*

- ☐ During your pregnancy
- ☐ Immediately after birth
- ☐ In the first few days
- ☐ In the first few weeks
- ☐ Only recently
- ☐ Not quite yet

**F2 Does your baby have any health problems now?**

☐ Yes ☐ No **If Yes**, *please give details* ____________________

**F3 How would you describe your baby as he or she is now?** *Please read the list and circle as many words as you like which best describe your baby.*

| | | | |
|---|---|---|---|
| placid | stubborn | cuddly | unresponsive |
| grizzly | active | angry | alert |
| responsive | demanding | sociable | inactive |
| withdrawn | happy | fretful | communicative |

**F4 Compared with other babies how would you say your baby is now?** *Please tick one box only*

☐ More difficult than average ☐ About average ☐ Easier than average

**F5 Parents' experiences with a baby vary a great deal.**

**How have the following been for you during the months since your baby was born?**
*Please tick one for each line*

| | Very difficult | Quite difficult | Not very difficult | Not at all difficult | Does not apply |
|---|---|---|---|---|---|
| Understanding what your baby needs | ☐ | ☐ | ☐ | ☐ | ☐ |
| Tiredness and lack of sleep | ☐ | ☐ | ☐ | ☐ | ☐ |
| The changes in your partner relationship | ☐ | ☐ | ☐ | ☐ | ☐ |
| Getting everything done | ☐ | ☐ | ☐ | ☐ | ☐ |
| Feeling you are doing the right thing for your baby | ☐ | ☐ | ☐ | ☐ | ☐ |
| Organising family life | ☐ | ☐ | ☐ | ☐ | ☐ |
| Adjusting to having a young baby around | ☐ | ☐ | ☐ | ☐ | ☐ |
| Loss of independence | ☐ | ☐ | ☐ | ☐ | ☐ |

**F6 Has your baby ever suffered from any of the following problems?**

- [ ] Sickness or vomiting
- [ ] Urinary tract infection
- [ ] Diarrhoea
- [ ] Colic / painful wind
- [ ] Constipation
- [ ] Thrush
- [ ] Chest problems / infection
- [ ] Not gaining enough weight
- [ ] Ear problems / infection
- [ ] Gaining too much weight
- [ ] Something else *Please give details* ________________

**F7 Since your baby was born…** *Please tick and write how many times*

| Including routine appointments, has your baby? | Roughly how many times? |
|---|---|
| ☐ Seen a health visitor | ☐ |
| ☐ Been taken to a GP (family doctor) | ☐ |
| ☐ Been taken to a regular baby clinic | ☐ |
| ☐ Been taken to a hospital emergency department (A&E) | ☐ |
| ☐ Been taken to a hospital outpatient clinic | ☐ |
| ☐ Stayed overnight in hospital | ☐ |

**F8 How much do you worry about your child's health and development?**
*Please tick one box only*

☐ A great deal ☐ Quite a lot ☐ Not very much ☐ Not at all

## Section G: Your Health and Wellbeing

**G1 Did you experience any of the following 10 days, 1 month, and 3 months after the birth of your baby?** *Please tick all that apply*

| | 10 days after the birth | 1 month after the birth | 3 months after the birth |
|---|---|---|---|
| 'The blues' | ☐ | ☐ | ☐ |
| Painful stitches or wound | ☐ | ☐ | ☐ |
| Depression | ☐ | ☐ | ☐ |
| Leaking urine *(stress incontinence)* | ☐ | ☐ | ☐ |
| Fatigue / severe tiredness | ☐ | ☐ | ☐ |
| Anxiety | ☐ | ☐ | ☐ |
| Sleep problems (not related to the baby) | ☐ | ☐ | ☐ |
| 'Flash-backs' to the labour or birth | ☐ | ☐ | ☐ |
| Relationship problems | ☐ | ☐ | ☐ |
| Difficulties in concentrating | ☐ | ☐ | ☐ |
| Other *Please give details* ________________ | ☐ | ☐ | ☐ |
| None of these | ☐ | ☐ | ☐ |

**G2 Overall, how have you felt physically during the last few days?** *Please tick one box only*

- ☐ 1 Very well
- ☐ 2 Quite well
- ☐ 3 Tired and uncomfortable
- ☐ 4 Exhausted all the time
- ☐ 5 Very ill

**G3 We would like to know how you have been feeling in the last week.**
Please circle any of the words below which describe how you have been feeling in the last 7 days.
*Circle as many as you wish*

| | | | |
|---|---|---|---|
| calm | tense | relaxed | upset |
| irritable | happy | miserable | excited |
| confident | drained | fulfilled | nervous |
| detached | contented | low | optimistic |
| cheerful | angry | energetic | impatient |
| restless | lighthearted | worried | satisfied |

Are there any other words you would like to add? ______________________________

______________________________________________

**G4 The following questions deal with your feelings. How have you felt over the LAST 7 DAYS, not just today.** *Please circle one box for each row*

| | | | | |
|---|---|---|---|---|
| I have been able to laugh and see the funny side of things | As much as I always could | Not quite so much now | Definitely not as much now | Not at all |
| I have looked forward with enjoyment to things | As much as I ever did | Rather less than I used to | Definitely less than I used to | Hardly at all |
| I have blamed myself unnecessarily when things went wrong | Yes, most of the time | Yes, some of the time | Not very often | No, never |
| I have been anxious or worried for no good reason | No, not at all | Hardly ever | Yes, sometimes | Yes, often |
| I have felt scared or panicky for no very good reason | Yes, quite a lot | Yes, sometimes | No, hardly ever | No, not at all |
| Things have been getting on top of me | Yes, most of the time | Yes, sometimes | No, hardly ever | No, not at all |
| I have been so unhappy, I have had difficulty sleeping | Yes, most of the time | Yes, sometimes | Not very often | No, not at all |
| I have felt sad and miserable | Yes, most of the time | Yes, sometimes | Not very often | No, not at all |
| I have been so unhappy that I have been crying | Yes, most of the time | Yes, quite often | Only occasionally | No, never |
| The thought of harming myself has occurred to me | Yes, most of the time | Sometimes | Hardly ever | Never |

## Section H: Your future plans

**H1 Are you doing any paid work at the moment?** *Please tick one box only*

- ☐ Yes
- ☐ On paid maternity leave ***Please go to question H4***
- ☐ On unpaid maternity leave ***Please go to question H4***
- ☐ No **If No, *go to question H4***

**H2 If you are working, how many hours do you work on average each week?**

☐☐ hours

**H3 If you are working, what age was your baby when you returned to work?**

☐☐ days **OR** ☐☐ weeks

**H4 Do you intend to start or return to work within the next year?**

☐ Yes ☐ No ☐ Don't know / not sure ☐ Have returned to work already

How old will your baby be when you return to work? ☐☐ days **OR** ☐☐ weeks

How many hours per week do you intend to work? ☐☐ hours

**H5 If you are working or plan to return to work how is your baby to be cared for while you are at work?** *Please tick all that apply*

- ☐ Childminder
- ☐ Nanny
- ☐ Nursery
- ☐ Husband or partner
- ☐ Friend
- ☐ Other person *Please give details* ______________________
- ☐ Not yet decided
- ☐ Does not apply

**H6 If you have returned to work or plan to return to work is this because…..**
*Please tick all that apply*

- ☐ You want to
- ☐ You need the money
- ☐ Work is really important to you
- ☐ You are sharing childcare with your partner
- ☐ You always planned to
- ☐ You have no choice
- ☐ You need to for your career
- ☐ You are not working or planning to return to work in the next year

## Section J: Your Lifestyle

**J1 Have you ever smoked tobacco cigarettes?**

☐ Yes ☐ No **If No, *please go to question J5***

**J2 How often did you smoke tobacco cigarettes during each of the following time periods?**
*(Please tick one box for each row)*

| | Daily | Less than daily, but at least once a week | Less than weekly, but at least once a month | Less than monthly | Not at all |
|---|---|---|---|---|---|
| In the 3 months before you became pregnant | ☐ | ☐ | ☐ | ☐ | ☐ |
| In the first 3 months of your pregnancy | ☐ | ☐ | ☐ | ☐ | ☐ |
| In the second 3 months of your pregnancy | ☐ | ☐ | ☐ | ☐ | ☐ |
| In the final 3 months of your pregnancy | ☐ | ☐ | ☐ | ☐ | ☐ |
| In the first 3 months after your baby was born | ☐ | ☐ | ☐ | ☐ | ☐ |
| How often do you smoke tobacco cigarettes now | ☐ | ☐ | ☐ | ☐ | ☐ |

**J3 Did you smoke tobacco cigarettes after you found out you were pregnant?**

☐ Yes ☐ No

**J4 After you found out you were pregnant, did you do any of the following?**
*Please tick all that apply*

☐ I stopped smoking temporarily (less than one month)
☐ I stopped smoking temporarily (more than one month)
☐ I cut down the number of cigarettes I smoked
☐ I used nicotine replacement therapy
(e.g. patch, gum, lozenges, nose spray, inhaler, microtabs)
☐ I used an electronic cigarette or vaping device
☐ Other *Please give details* ____________________

**J5 Have you ever used an electronic cigarette or vaping device?**

☐ Yes ☐ No **If No, *please go to question J9***

**J6 How often did you use an electronic cigarette or vaping device during each of the following time periods?** *(Please tick one box for each row)*

| | Daily | Less than daily, but at least once a week | Less than weekly, but at least once a month | Less than monthly | Not at all |
|---|---|---|---|---|---|
| In the 3 months before you became pregnant | ☐1 | ☐1 | ☐1 | ☐1 | ☐1 |
| In the first 3 months of your pregnancy | ☐1 | ☐1 | ☐1 | ☐1 | ☐1 |
| In the second 3 months of your pregnancy | ☐1 | ☐1 | ☐1 | ☐1 | ☐1 |
| In the final 3 months of your pregnancy | ☐1 | ☐1 | ☐1 | ☐1 | ☐1 |
| In the first 3 months after your baby was born | ☐1 | ☐1 | ☐1 | ☐1 | ☐1 |
| How often do you use an electronic cigarette or vaping device now | ☐1 | ☐1 | ☐1 | ☐1 | ☐1 |

**J7 Did you use an electronic cigarette or vaping device after you found out you were pregnant?**

☐1 Yes ☐2 No

**J8 When you used an electronic cigarette or vaping device, did it contain nicotine?**

☐1 Always ☐2 Sometimes ☐3 No, never ☐4 I don’t know

**J9 During your pregnancy, did any of the people you lived with smoke tobacco cigarettes?**

☐1 Yes, I lived with my partner who smoked
☐2 Yes, someone else I lived with smoked
☐3 No, nobody else who I lived with smoked
☐4 Does not apply – I lived alone

**J10 Do any of the people who live with you now smoke tobacco cigarettes?**

☐1 Yes, I live with my partner who smokes
☐2 Yes, someone else I live with smokes
☐3 No, nobody else who I live with smokes
☐4 Not applicable – I live alone with my baby

## Section K: You and Your Household

Please answer as many of these questions as you can. This will help us to describe the women taking part in this survey and show whether the care offered to women is the same regardless of their background or circumstances.

**K1 How old are you now?**

☐☐ years

**K2 How old were you when you left full-time education?**

☐1 16 years or less ☐2 17 or 18 years ☐3 19 years or over ☐4 Still in full-time education

**K3 Have you had a previous pregnancy (<u>before</u> this one)?**

- [ ] Yes
- [ ] No

**K4 Have you given birth before having this baby?**

- [ ] Yes
- [ ] No

**If Yes**, how many babies have you given birth to *<u>before</u>* this pregnancy?

[ ][ ] babies

**K5 Which of the following people live with you?** *Please tick all that apply*

- [ ] Your baby / children
- [ ] Husband / partner
- [ ] Other family members
- [ ] Other people in your household

**K6 In which country were you born?** ____________________

If you were born outside the United Kingdom, what year did you come to the UK? [ ][ ][ ][ ]

**K7 What is your ethnic group?** *Please tick one box only*

**WHITE**

- [ ] English / Welsh / Scottish / Northern Irish / British
- [ ] Irish
- [ ] Gypsy or Irish Traveller
- [ ] Any other White background

If other, please write in box

[ ]

**MIXED**

- [ ] White and Black Caribbean
- [ ] White and Black African
- [ ] White and Asian
- [ ] Any other mixed / multiple ethnic background

If other, please write in box

[ ]

**ASIAN OR ASIAN BRITISH**

- [ ] Indian
- [ ] Pakistani
- [ ] Bangladeshi
- [ ] Chinese
- [ ] Any other Asian background

If other, please write in box

[ ]

**BLACK OR BLACK BRITISH**

- [ ] African
- [ ] Caribbean
- [ ] Any other Black / African / Caribbean

If other, please write in box

[ ]

**OTHER ETHNIC GROUP**

- [ ] Arab
- [ ] Any other ethnic group

If other, please write in box

[ ]

**If there is anything else you would like to tell us about your care while you were pregnant or since you have had your baby, please add your comments here, or use the back page.**

**Continue over the page, if you would like to.**

**If there is anything else you would like to tell us about your care while you were pregnant or since you have had your baby, please add your comments here.**

**Was there anything else you meant to go back and complete later?**

Please check you haven't missed any pages. If the survey has raised issues or questions of concern you may wish to contact your family doctor (GP) or health visitor.

**Thank you very much for your help**

**Please return the questionnaire in the envelope provided or respond online. If you have any queries about the questionnaire or you would like to know more about the research please contact:**

Emily Savage McGlynn, National Perinatal Epidemiology Unit
University of Oxford, Old Road Campus, Headington, Oxford. OX3 7LF
Tel. 01865 289700

emily.savage-mcglynn@npeu.ox.ac.uk

UNIVERSITY OF OXFORD
