# Supplementary material for: Methods to increase response rates to a population-based maternity survey: a comparison of two pilot studies
Source: BMC Med Res Methodol. 2019 Mar 20;19:65. doi: 10.1186/s12874-019-0702-3 (PMC6425628; doi:10.1186/s12874-019-0702-3)
Supplement: Supplementary file 2 — You and Your Baby: A national survey of health and care. Pilot survey 2 questionnaire. (PDF 1852 kb) [file 12874_2019_702_MOESM2_ESM.pdf]

You & your Baby

A national survey of health and care

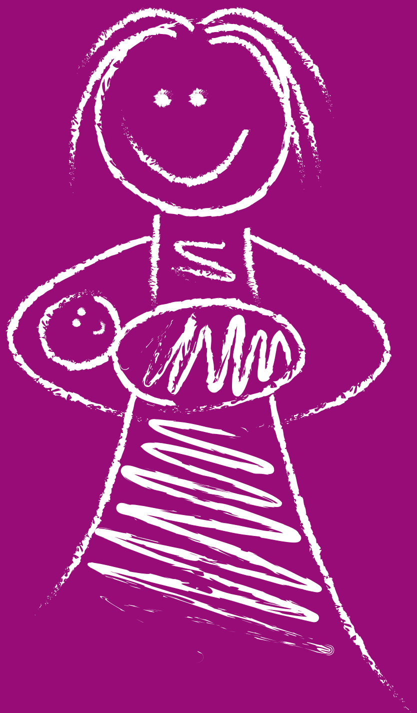

This survey is about you and your baby, your health and wellbeing, your experiences of having a baby, and the care that you both received.

By taking part, the information you share with us will be used to help improve the care that mums and babies receive.

For most questions, make a cross clearly inside one box ☒ or write clearly in the space provided. For some questions, where indicated, you may mark more than one box.

You can complete this survey online by going to:

www.npeu.ox.ac.uk/maternity-surveys

(ID number and password in the small box below)

If you would like to talk to us about the study, please call us on:

Freephone 0808 252 4566

PRUMHC
POLICY RESEARCH UNIT - MATERNAL HEALTH & CARE

npeu
National Perinatal Epidemiology Unit

UNIVERSITY OF OXFORD

You and Your Baby Questionnaire 2a - 2.0 (4.5.17) - REC Ref: 16/YH/0412

Part A: Your pregnancy...

If you have been pregnant before, please only think about your most recent pregnancy when answering these questions.

A1. Did you give birth to a single baby, twins or more babies in your most recent pregnancy?

☐ A single baby ☐ Twins ☐ Triplets, quads or more

A2. If you had a single baby, is your baby…?

☐ A boy **OR** ☐ A girl

If you had a multiple birth, did you have…?

☐ All boys **OR** ☐ All girls **OR** ☐ boy and girl twins, triplets or more

Note: *If you had more than one baby in your most recent pregnancy, please fill in the rest of the questionnaire about the baby who was born first as a result of this pregnancy*

A3. When was your baby born?

D D / M M / Y Y (date / month / year)

A4. Roughly how many weeks pregnant were you when your baby was born?

☐☐ weeks

A5. How much did your baby weigh at birth?

Grams ☐☐☐☐ **OR** Pounds ☐☐ and ounces ☐☐

A6. Did you plan to get pregnant with this baby?

☐ Yes ☐ No

A7. Roughly how many weeks pregnant were you when you had your pregnancy 'booking'?

(the appointment where you were given your hand held pregnancy records/notes)

☐☐ weeks

A8. At the time of your pregnancy booking or a few weeks later were you asked about your emotional and mental health?

☐ Yes ☐ No ☐ Not sure / Don't know

A9. Did you have a mental health problem during your pregnancy?

☐ Yes ☐ No ☐ Not sure / Don't know

If Yes, was this? *Please tick all that apply*

☐ Anxiety?
☐ Depression?
☐ Other condition? Please give details: ____________________

Was this a problem you had before this pregnancy?

☐ Yes ☐ No

Part B: Pregnancy check-ups...

A 'check-up' is any contact with a midwife or a doctor to check the progress of your pregnancy. This usually includes having your blood pressure and urine checked.

Please ignore other appointments that did not include these things, such as a visit for a scan or a blood test only.

B1. Roughly how many check-ups did you have at each of these places?

Please write in the number for each

Local clinic ☐☐ Children's centre ☐☐ GP surgery ☐☐

Hospital clinic ☐☐ At home ☐☐

B2. During your pregnancy how many ultrasound scans (showing your baby on a screen) did you have in total?

☐☐

B3. Altogether, how many different midwives looked after you during your pregnancy?

Tick one box only

☐ One ☐ Two ☐ Three ☐ Four ☐ Five or more

Part C: Your labour and the birth of your baby...

C1. Before you went into labour or had your baby did you have any particular worries about the labour and birth? *Please tick one box for each line*

	Very worried	Quite worried	Not very worried	Not at all worried
Not knowing when I would go into labour	☐	☐	☐	☐
Getting to the hospital in time	☐	☐	☐	☐
Having to be induced	☐	☐	☐	☐
Having a long labour	☐	☐	☐	☐
Pain and discomfort of labour	☐	☐	☐	☐
Getting effective pain relief	☐	☐	☐	☐
Not knowing how long labour would take	☐	☐	☐	☐
Having a forceps or ventouse delivery	☐	☐	☐	☐
Embarrassment	☐	☐	☐	☐
Needing a caesarean	☐	☐	☐	☐
Other, please give details: ______	☐	☐	☐	☐

C2. Where was your baby born? *Please tick one box only*

- ☐ At home
- ☐ In a midwife-led unit or birth centre separate from hospital
- ☐ In hospital, in a midwife-led unit *(in the same hospital, but separate from the consultant-led unit)*
- ☐ In hospital, in a consultant-led unit (obstetric unit)
- ☐ Other, please give details: ______________________

C3. Did you have a labour?

☐ Yes ☐ No **If No, *please go to Question D1***

C4. How did your labour start? *Please tick all that apply*

- ☐ It started naturally
- ☐ I had one or more membrane sweeps
- ☐ I was given a vaginal gel or pessary to induce my labour
- ☐ My waters were broken by a doctor or a midwife (amniotomy)
- ☐ I was given a drip (in my hand or arm) to induce my labour

C5. During your labour, did you use any of these for pain relief? *Please tick all that apply*

- ☐ Gas and air (breathing through a mask)
- ☐ Injection of pethidine or a similar painkiller
- ☐ Epidural or similar (injection in your back)
- ☐ Other, please give details: ______________________

Part D: The birth of your baby...

D1. How was your baby born? *Please tick one box only*

- ☐ Normal (vaginal) birth
- ☐ Delivery using forceps
- ☐ Delivery using vacuum cap on the baby's head (ventouse)
- ☐ A caesarean (through a cut in the abdomen)

If your baby was born by **caesarean** was this... *Please tick one box only*

- ☐ Planned and carried out before you went into labour?
- ☐ Planned, but carried out after you had gone into labour?
- ☐ The result of an unforeseen problem during your labour?

If you had a caesarean birth this time, please go to Question D4

D2. If you had a vaginal birth, while your baby was being born did you have an episiotomy (cut) to assist with the delivery of the baby?

☐ Yes ☐ No ☐ Don't know / Can't remember

D3. If you had a vaginal birth, while your baby was being born did you have a tear (not a deliberate cut)? *Please tick one box only*

- ☐ No
- ☐ Yes, a tear that did not need stitches
- ☐ Yes, a tear that needed stitches
- ☐ Yes, a serious tear which involved my back passage (third or fourth degree tear)
- ☐ Don't know / Can't remember

Please answer the following questions if you had a vaginal birth OR a caesarean.

D4. Women have different experiences of labour and birth. How much do you agree or disagree with the following statements? *Please circle one number on each row*

	Strongly Agree	Agree	Neither agree or disagree	Disagree	Strongly Disagree
I came through childbirth virtually unscathed	1	2	3	4	5
I thought my labour was excessively long	1	2	3	4	5
The delivery room staff encouraged me to take decisions about how I wanted my birth to progress	1	2	3	4	5
I felt very anxious during my labour and birth	1	2	3	4	5
I felt well supported by staff during my labour and birth	1	2	3	4	5
The staff communicated well with me during labour	1	2	3	4	5
I found giving birth a distressing experience	1	2	3	4	5
I felt out of control during my birth experience	1	2	3	4	5
I was not distressed at all during labour	1	2	3	4	5
The delivery room was clean and hygienic	1	2	3	4	5

D5. Altogether, how many different midwives looked after you during your labour and/or the birth of your baby? *Please tick one box only*

☐ One ☐ Two ☐ Three ☐ Four ☐ Five or more

D6. Had you met any of the midwives before you went into labour or gave birth?
Please tick one box only

☐ All of them ☐ Some of them ☐ None of them

D7. How soon after birth did you…

Hold your baby?	☐☐ minutes	☐☐ hours	☐☐ days
Have skin-to-skin contact with your baby	☐☐ minutes	☐☐ hours	☐☐ days

(By skin-to-skin contact we mean you were holding the baby so that his/her bare skin was next to your bare skin)

D8. Overall, how do you feel your labour and birth went? *Please tick one box only*

☐ Worse than you expected
☐ More or less as you expected
☐ Better than you expected

D9. We would like to know how you feel you were looked after during your labour and birth. Please circle any of the words below which describe the staff you saw during labour. *Circle as many as you wish*

rushed	humorous	insensitive	kind
considerate	unhelpful	supportive	offhand
rude	warm	inconsiderate	polite
sensitive	bossy	informative	condescending

Part E: After the birth of your baby...

E1. Did you stay in a maternity unit or hospital after your baby was born?

☐ Yes ☐ No

If Yes, how long did you stay in the maternity unit (or hospital) after your baby was born?

☐☐ hours **OR** ☐☐ days

After birth some babies need specialist care, e.g. help with breathing, and are admitted to a neonatal unit, sometimes called a Special Care Baby Unit (SCBU) or Neonatal Intensive Care Unit (NICU).

E2. Was your baby cared for in a neonatal unit at all?

☐ Yes ☐ No

If Yes, for how long?

☐☐ hours **OR** ☐☐ days **OR** ☐☐ weeks **OR** ☐☐ months
OR ☐ My baby is still being cared for in a neonatal unit

E3. After the birth of your baby, how many times in total did you…

… see a midwife or maternity support worker at home? ☐☐ times
… see a midwife or maternity support worker at a drop in clinic? ☐☐ times
… have contact with a midwife or maternity support worker by phone? ☐☐ times

E4. How many different midwives in total visited you at home after your baby was born?
Please tick one box only

☐ One ☐ Two ☐ Three or more

E5. Had you met any of these midwives before you had your baby? *Please tick one box only*

☐ All of them ☐ Some of them ☐ None of them

E6. Parents' experiences with a baby vary a great deal. How have the following been for you during the months since your baby was born? *Please tick one box for each row*

	Very difficult	Quite difficult	Not very difficult	Not at all difficult	Does not apply
Understanding what your baby needs	☐	☐	☐	☐	☐
Tiredness and lack of sleep	☐	☐	☐	☐	☐
The changes in your partner relationship	☐	☐	☐	☐	☐
Getting everything done	☐	☐	☐	☐	☐
Feeling you are doing the right thing for your baby	☐	☐	☐	☐	☐
Organising family life	☐	☐	☐	☐	☐
Adjusting to having a young baby around	☐	☐	☐	☐	☐
Loss of independence	☐	☐	☐	☐	☐

Section F: Feeding your baby...

Note. When we ask about 'breastfeeding' we also mean 'giving your baby expressed breast milk'

F1. Did you ever try to breastfeed your baby, even if it was only once?

☐ Yes ☐ No **If No, *please go to Question F3***

F2. How old was your baby when he or she was last given breast milk?

☐☐ days **OR** ☐☐ weeks **OR** ☐☐ months

OR ☐ My baby is still being given breast milk

F3. In the first few days after the birth how was your baby fed? *Please tick one box only*

☐ Breast milk (or expressed breast milk) only
☐ Both breast and formula (bottle) milk
☐ Formula (bottle) milk only
☐ Not sure

F4. Would you have liked more help from a health professional with breastfeeding your baby?

☐ Yes ☐ No

F5. How is your baby fed now? (in the last 24 hours) *Please tick one box only*

☐ Formula (bottle) milk only
☐ Breast milk (or expressed breast milk) only
☐ Both breast and formula (bottle) milk
☐ Other, please give details: ____________________

Part G: Your baby...

G1. When did you first feel your baby really belonged to you? *Please tick one box only*

- ☐ During your pregnancy
- ☐ Immediately after birth
- ☐ In the first few days
- ☐ In the first few weeks
- ☐ Only recently
- ☐ Not quite yet

G2. How would you describe your baby as he or she is now? *Please read the list and **circle** as many words as you like which best describe your baby*

placid	stubborn	cuddly	unresponsive
grizzly	active	angry	alert
responsive	demanding	sociable	inactive
withdrawn	happy	fretful	communicative

G3. Compared to other babies, how would you describe your baby? *Please tick one box only*

☐ More difficult than most ☐ About average ☐ Easier than most

G4. Does your baby have any health problems now?

☐ No ☐ Yes, please give details: ______________________

G5. How much do you worry about your child's health and development?

Please tick one box only

☐ A great deal ☐ Quite a lot ☐ Not very much ☐ Not at all

Part H: Your health and wellbeing...

H1. Overall, how have you felt physically during the last few days? *Please tick one box only*

☐ Very well ☐ Quite well ☐ Quite unwell ☐ Very unwell

H2. Overall, how tired have you felt during the last few days? *Please tick one box only*

☐ Not very tired ☐ Quite tired ☐ Very tired ☐ Exhausted all the time

H3. Over the last 2 weeks, how often have you been bothered by the following problems?

Please circle one answer for each row

Feeling nervous, anxious, or on edge	Not at all	Several days	More than half the days	Nearly every day
Not being able to stop or control worrying	Not at all	Several days	More than half the days	Nearly every day

H4. Did you have a postnatal check-up of your own health with your GP / family doctor (usually between 4–8 weeks after the birth)?

☐ Yes ☐ No

H5. Since your baby was born have you been asked about your emotional and mental health by a health professional?

☐ Yes ☐ No ☐ Don't know / Can't remember

H6. Did you experience any of the following after the birth of your baby?

Please tick all that apply

	10 days after the baby's birth	1 month after the baby's birth	1 month after the baby's birth
'The blues'	☐	☐	☐
Painful stitches or wound	☐	☐	☐
Depression	☐	☐	☐
Stress incontinence (leaking urine)	☐	☐	☐
Fatigue / severe tiredness	☐	☐	☐
Anxiety	☐	☐	☐
Sleep problems (not related to the baby)	☐	☐	☐
'Flash-backs' to the labour or birth	☐	☐	☐
Relationship problems	☐	☐	☐
Difficulties in concentrating	☐	☐	☐
Other, please give details: ______________	☐	☐	☐
None of these	☐	☐	☐

H7. We would like to know how you have been feeling in the last week. *Please circle as many of the words below as you like which describe how you have been feeling in the last 7 days*

Calm	Tense	Relaxed	Upset
Irritable	Happy	Miserable	Excited
Confident	Drained	Fulfilled	Nervous
Exhausted	Contented	Low	Optimistic
Cheerful	Angry	Energetic	Impatient
Restless	Grateful	Worried	Satisfied

☐ None of these words describe how I have been feeling in the last seven days

Are there any other words that describe how you have been feeling?

H8. I generally feel good about myself: *Please circle one number only*

Not very true of me 1 2 3 4 5 6 7 Very true of me

H9. The following questions deal with your feelings. How have you felt over the last 7 days, not just today? *Please circle one answer for each row*

I have been able to laugh and see the funny side of things	As much as I always could	Not quite so much now	Definitely not so much now	Not at all
I have looked forward with enjoyment to things	As much as I ever did	Rather less than I used to	Definitely less than I used to	Hardly at all
I have blamed myself unnecessarily when things went wrong	Yes, most of the time	Yes, some of the time	Not very often	No, never
I have been anxious or worried for no good reason	No, not at all	Hardly ever	Yes, sometimes	Yes, very often
I have felt scared or panicky for no very good reason	Yes, quite a lot	Yes, sometimes	No, not much	No, not at all
Things have been getting on top of me	Yes, most of the time	Yes, sometimes	No, not very often	No, not at all
I have been so unhappy that I have had difficulty sleeping	Yes, most of the time	Yes, sometimes	Not very often	No, not at all
I have felt sad and miserable	Yes, most of the time	Yes, quite often	Not very often	No, not at all
I have been so unhappy that I have been crying	Yes, most of the time	Yes, quite often	Only occasionally	No, never
The thought of harming myself has occurred to me	Yes, quite often	Sometimes	Hardly ever	Never

Part I: Your experience of maternity care…

Women's experience of maternity care is different. How much do you agree with the following statements? Please tick one box for each row

I1. Thinking about my care *during pregnancy*…

	Strongly Agree	Agree	Neither agree or disagree	Disagree	Strongly Disagree
I felt I had the right number of antenatal checks with the midwife/doctor					
My care provider(s) gave me all the information I needed					
I always saw the same midwife/doctor for my antenatal checks					
Health professionals did not always talk to me in a way I could understand					
Antenatal appointments were too short to discuss any concerns about my pregnancy					
I was not involved enough in decisions about my antenatal care					
I was happy with the number of health professionals who cared for me during my pregnancy					
I was not given enough explanations about antenatal scans and tests					
I was not given enough information to make decisions about my antenatal care					
I would have liked more antenatal checks and scans					
During pregnancy, I did not feel well cared for by health professionals					
Overall, I was very pleased with the care I received in pregnancy					

I2. Thinking about my care *during labour and birth*…

	Strongly Agree	Agree	Neither agree or disagree	Disagree	Strongly Disagree
Staff communicated well with me during labour and birth	☐	☐	☐	☐	☐
I needed more staff support during labour and birth	☐	☐	☐	☐	☐
Everything was explained to me well during labour and birth	☐	☐	☐	☐	☐
I was treated as an individual by staff	☐	☐	☐	☐	☐
I was not involved enough in decisions about procedures that were carried out (e.g. breaking waters, caesarean section)	☐	☐	☐	☐	☐
Health professionals left me alone more than I would have liked	☐	☐	☐	☐	☐
I felt that my pain relief needs were not managed well	☐	☐	☐	☐	☐
I felt safe in the labour and birth environment	☐	☐	☐	☐	☐
The staff could have done more to help me to feel in control of my labour and birth	☐	☐	☐	☐	☐
I had confidence and trust in the staff caring for me	☐	☐	☐	☐	☐
I did not mind being looked after by midwives or doctors I had not met before	☐	☐	☐	☐	☐
I had the best possible care during labour and birth	☐	☐	☐	☐	☐

I3. Thinking about my *postnatal* care…

	Strongly Agree	Agree	Neither agree or disagree	Disagree	Strongly Disagree
I received enough care and attention from staff on the postnatal ward	☐	☐	☐	☐	☐
I stayed in hospital as long as I wanted after the birth	☐	☐	☐	☐	☐
I was treated as an individual by midwives/ doctors after the birth	☐	☐	☐	☐	☐
After I had given birth, health professionals treated me as though I was no longer important	☐	☐	☐	☐	☐
I had enough information from health professionals about how to care for my baby	☐	☐	☐	☐	☐
I was able to build a good relationship with the healthcare professionals I saw after coming home	☐	☐	☐	☐	☐
I was not given the advice and information I needed by health professionals after my baby was born	☐	☐	☐	☐	☐
There was not enough time to talk over my concerns with health professional	☐	☐	☐	☐	☐
I had all the checks I needed after the birth	☐	☐	☐	☐	☐
After the birth of my baby, I knew who to contact if I had questions or concerns	☐	☐	☐	☐	☐
The postnatal care I received did not meet the needs of me and my baby	☐	☐	☐	☐	☐
Overall I was very pleased with the quality of my postnatal care	☐	☐	☐	☐	☐

Part J: You and Your Household...

Please answer as many of these questions as you can. This will help us to describe the women taking part in this survey and show whether the care offered to women is the same regardless of their background or circumstances.

J1. Have you had a previous pregnancy (before this one)?

☐ Yes ☐ No **If No, *please go to Question J4***

J2. Have you given birth before having this baby?

☐ Yes ☐ No **If No, *please go to Question J4***

J3. How many babies have you given birth to before this pregnancy?

☐☐ babies

J4. How old are you now?

☐☐ years

J5. How old were you when left full-time education?

☐ 16 years or less ☐ 17 or 18 years ☐ 19 years or over ☐ Still in full-time education

J6. Which of the following people live with you?

- ☐ Your baby / other children
- ☐ Your spouse / civil partner / partner
- ☐ Other family members
- ☐ Other, please tell us who: ____________________

J7. In which country were you born?

____

J8. If you were born outside the United Kingdom, what year did you come to the UK?

☐☐☐☐

J9. What is your ethnic group? *Please tick one box only*

White:

- ☐ English / Welsh / Scottish / Northern Irish / British
- ☐ Irish
- ☐ Gypsy or Irish Traveller
- ☐ Any other White background

If other, please write in box:

Mixed:

- ☐ White & Black Caribbean
- ☐ White & Black African
- ☐ White & Asian
- ☐ Any other Mixed / multiple ethnic background

If other, please write in box:

Other ethnic group:

- ☐ Arab
- ☐ Any other ethinic background

If other, please write in box:

Asian or Asian British:

- ☐ **Indian**
- ☐ Pakistani
- ☐ Bangladeshi
- ☐ Chinese
- ☐ Any other Asian background

If other, please write in box:

Black or Black British:

- ☐ Caribbean
- ☐ African
- ☐ Any other Black/African/Caribbean background

If other, please write in box:

- ☐ **Not stated**

J10. Do you have a longstanding physical health problem or disability?

☐ Yes ☐ No

J11. Do you have a longstanding mental health problem?

☐ Yes ☐ No

J12. Do any of the problems or disabilities affect your day-to-day activities?

☐ Yes, definitely ☐ Yes, to some extent ☐ No ☐ Not applicable

J13. Would you mind telling us what your mental / physical health problem and / or disability is?

Is there anything else you would like to tell us about yourself, your baby or the care you have received?

Thank you

Was there anything else you meant to go back to and complete later?

Please check you haven't missed any pages.

If the survey has raised issues or questions of concern you may wish to contact your family doctor (GP) or health visitor.

Please return the questionnaire in the FREEPOST envelope provided. If you have any queries about the questionnaire or you would like to know more about the research please contact the research team:

Maria Quigley and Sian Harrison

National Perinatal Epidemiology Unit
University of Oxford
Old Road Campus
Headington
Oxford OX3 7LF

Tel: FREEPHONE 0808 252 4566
Email: sian.harrison@npeu.ox.ac.uk

You & your Baby
A national survey of health and care
