# Supplementary material for: Methods to increase response rates to a population-based maternity survey: a comparison of two pilot studies
Source: BMC Med Res Methodol. 2019 Mar 20;19:65. doi: 10.1186/s12874-019-0702-3 (PMC6425628; doi:10.1186/s12874-019-0702-3)
Supplement: Supplementary file 3 [file 12874_2019_702_MOESM3_ESM.docx]

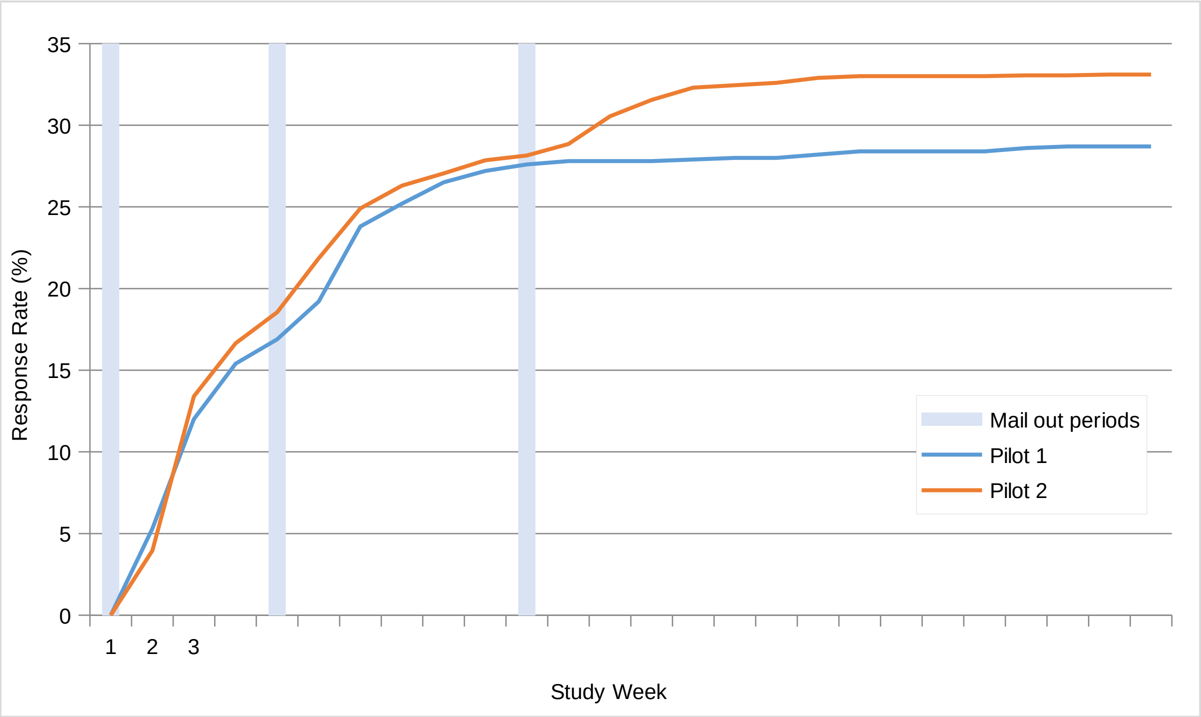

35
30
25
20
15
10
5
0
Response Rate (%)
1
2
3
Study Week
Mail out periods
Pilot 1
Pilot 2
