# Supplementary material for: Methods to increase response rates to a population-based maternity survey: a comparison of two pilot studies
Source: BMC Med Res Methodol. 2019 Mar 20;19:65. doi: 10.1186/s12874-019-0702-3 (PMC6425628; doi:10.1186/s12874-019-0702-3)
Supplement: Supplementary file 4 [file 12874_2019_702_MOESM4_ESM.docx]

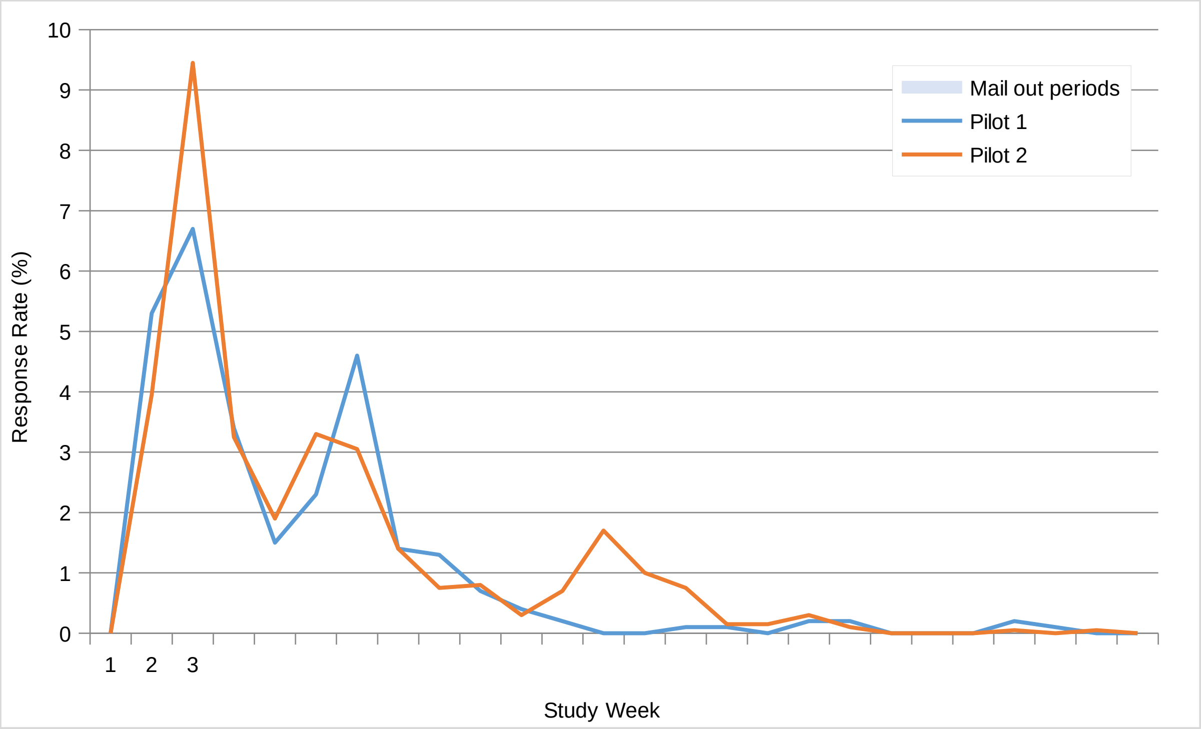

Mail out periods
Pilot 1
Pilot 2
Response Rate (%)
Study Week
10
9
8
7
6
5
4
3
2
1
0
1
2
3
